# Evaluation of the redox state in mouse organs following radon inhalation

**DOI:** 10.1093/jrr/rraa129

**Published:** 2021-01-27

**Authors:** Takahiro Kataoka, Norie Kanzaki, Akihiro Sakoda, Hina Shuto, Junki Yano, Shota Naoe, Hiroshi Tanaka, Katsumi Hanamoto, Hiroaki Terato, Fumihiro Mitsunobu, Kiyonori Yamaoka

**Affiliations:** Graduate School of Health Sciences, Okayama University, 5-1 Shikata-cho 2-chome, Kita-ku, Okayama-shi, Okayama 700-8558, Japan; Ningyo-toge Environmental Engineering Center, Japan Atomic Energy Agency, 1550 Kamisaibara, Kagamino-cho, Tomata-gun, Okayama 708-0698, Japan; Ningyo-toge Environmental Engineering Center, Japan Atomic Energy Agency, 1550 Kamisaibara, Kagamino-cho, Tomata-gun, Okayama 708-0698, Japan; Graduate School of Health Sciences, Okayama University, 5-1 Shikata-cho 2-chome, Kita-ku, Okayama-shi, Okayama 700-8558, Japan; Graduate School of Health Sciences, Okayama University, 5-1 Shikata-cho 2-chome, Kita-ku, Okayama-shi, Okayama 700-8558, Japan; Graduate School of Health Sciences, Okayama University, 5-1 Shikata-cho 2-chome, Kita-ku, Okayama-shi, Okayama 700-8558, Japan; Ningyo-toge Environmental Engineering Center, Japan Atomic Energy Agency, 1550 Kamisaibara, Kagamino-cho, Tomata-gun, Okayama 708-0698, Japan; Graduate School of Health Sciences, Okayama University, 5-1 Shikata-cho 2-chome, Kita-ku, Okayama-shi, Okayama 700-8558, Japan; Advanced Science Research Center, Okayama University, 5-1 Shikata-cho 2-chome, Kita-ku, Okayama-shi, Okayama 700-8558, Japan; Graduate School of Medicine Dentistry and Pharmaceutical Sciences, Okayama University, 5-1 Shikata-cho 2-chome, Kita-ku, Okayama-shi, Okayama 700-8558, Japan; Graduate School of Health Sciences, Okayama University, 5-1 Shikata-cho 2-chome, Kita-ku, Okayama-shi, Okayama 700-8558, Japan

**Keywords:** radon, redox state, oxidative stress, antioxidative function, principal component analysis

## Abstract

Radon inhalation activates antioxidative functions in mouse organs, thereby contributing to inhibition of oxidative stress-induced damage. However, the specific redox state of each organ after radon inhalation has not been reported. Therefore, in this study, we evaluated the redox state of various organs in mice following radon inhalation at concentrations of 2 or 20 kBq/m^3^ for 1, 3 or 10 days. Scatter plots were used to evaluate the relationship between antioxidative function and oxidative stress by principal component analysis (PCA) of data from control mice subjected to sham inhalation. The results of principal component (PC) 1 showed that the liver and kidney had high antioxidant capacity; the results of PC2 showed that the brain, pancreas and stomach had low antioxidant capacities and low lipid peroxide (LPO) content, whereas the lungs, heart, small intestine and large intestine had high LPO content but low antioxidant capacities. Furthermore, using the PCA of each obtained cluster, we observed altered correlation coefficients related to glutathione, hydrogen peroxide and LPO for all groups following radon inhalation. Correlation coefficients related to superoxide dismutase in organs with a low antioxidant capacity were also changed. These findings suggested that radon inhalation could alter the redox state in organs; however, its characteristics were dependent on the total antioxidant capacity of the organs as well as the radon concentration and inhalation time. The insights obtained from this study could be useful for developing therapeutic strategies targeting individual organs.

## INTRODUCTION

Epidemiological studies in Europe [1] and North America [2] have indicated that indoor radon exposure causes lung cancer. The adverse health effects of radon progeny have also been reported [3]. Moreover, analysis of immune function by detecting lymphocyte subsets in the peripheral blood of residents living in the vicinity of radon-rich hot springs showed that radon-rich hot springs could alter the proportions of lymphocyte subsets and possibly affect immunologic functions [4]. However, the total amount of inhaled radon was much lower in residents living near radon-rich hot springs than in the former indoor radon exposure studies. Thus, the health effects of radon can vary depending on the total amount of inhaled radon.

Radon therapy was shown to alleviate the symptoms of osteoarthritis [5] and bronchial asthma [6] through the activation of antioxidative functions. A meta-analysis of controlled clinical trials of radon therapy revealed positive effects of radon therapy in patients with pain due to rheumatic diseases [7]. However, the radon concentration used by a study conducted in Montana was about 20 times higher than that used by a study in Misasa (~2000 Bq/m^3^) [8].

Furthermore, doctors make decisions regarding treatment methods based on their experiences because the mechanisms through which radon exerts its beneficial effects are still unclear. In addition, examination of antioxidative functions in organs can reveal new indications for radon therapy. To this end, our previous study showed that radon inhalation increases superoxide dismutase (SOD) in mouse organs [9]. This activation induced by radon inhibits several types of oxidative damage, including oxidative damage to the liver [10, 11], kidneys [12], brain [13] and colon [14], in mice. Moreover, we previously found that manganese SOD was induced in the brain by oxidative stress following radon inhalation [15]. These studies indicated that radon inhalation may alleviate oxidative stress-induced diseases by activating antioxidative function in organs induced by moderate oxidative stress.

Therefore, in this study, we aimed to evaluate the effects of inhalation time, radon concentrations and the redox state in different organs of mice following radon inhalation. Our findings revealed the potential of radon inhalation to alter the redox state of the organs and suggested that the therapeutic effects of radon inhalation were likely related to alterations in the antioxidative functions of organs.

## MATERIALS AND METHODS

### Animals

Male BALB/c mice (8 weeks old) were obtained from CLEA Japan Inc. (Tokyo, Japan). Animals were housed under standard environmental conditions, i.e. temperature 24 ± 2 °C and a preset light–dark cycle of 12:12 h. Ethics approval was obtained from the Animal Care and Use Committee of Okayama University.

### Experimental procedures

Experimental mice were randomly categorized into seven groups of seven animals each. The control group received a sham inhalation only, whereas the radon group was treated with radon inhalation at concentrations of 2 or 20 kBq/m^3^ for 1, 3 or 10 days. Mice were euthanatized using CO_2_. After euthanasia, blood was drawn from the heart, and the brains, lungs, hearts, livers, stomachs, pancreases, kidneys, small intestines and large intestines were removed quickly. Samples were stored at −80°C until analysis. Tissue samples were used to assess levels of SOD, catalase (CAT), total glutathione (t-GSH), lipid peroxide (LPO) and hydrogen peroxide (H_2_O_2_).

### Biochemical assays

For SOD, CAT, t-GSH, H_2_O_2_ and LPO assays, samples were homogenized in 10 mM phosphate-buffered saline (PBS; pH 7.4), and homogenates were used for analyses. The SOD activity and t-GSH and LPO levels were measured following the method described in our previous study [16].

CAT activity was measured using a Catalase Assay Kit (Cayman Chemical, MI, USA), which uses a method based on the reaction of the enzyme with methanol in the presence of an optimal concentration of H_2_O_2_. The formaldehyde produced was measured colorimetrically with 4-amino-3-hydrazino-5-mercapto-1,2,4-triazole (Purpald) as the chromogen; Purpald specifically forms a bicyclic heterocycle with aldehydes, which changes from colorless to a purple color upon oxidation [17, 18]. Then, the absorbance was read at 540 nm using a plate reader.

H_2_O_2_ levels were measured using an Oxiselect Hydrogen Peroxide/Peroxidase Assay Kit (Cell Biolabos, Inc., San Diego, CA, USA). Briefly, in the presence of peroxidase, the probe reacted with H_2_O_2_ in a 1:1 stoichiometry to produce a bright pink-colored product, which could be measured at 540 nm and was directly proportional to the H_2_O_2_ levels in the sample.

### Statistical analyses

Data are presented as means ± standard errors of the means. The statistical significance of biochemical assays was determined using one-way analysis of variance following Tukeyʼs test for multiple comparisons. Differences with *P* values < 0.05 were considered statistically significant. Principal component analysis (PCA) was performed using R software. The first principal component (PC1) is required to have the largest possible variance, whereas the second component (PC2) is computed under the constraint of being orthogonal to the first component and has the largest possible inertia [19]. The cumulative contribution and the contribution ratio of each indicator to each axis (PC1, PC2) were estimated for each PCA. Correlation coefficients were determined using Excel. Pearson’s tests were performed to determine the differences among groups.

## RESULTS

### Evaluation of the redox state of organs using PCA

**Fig. 1.**
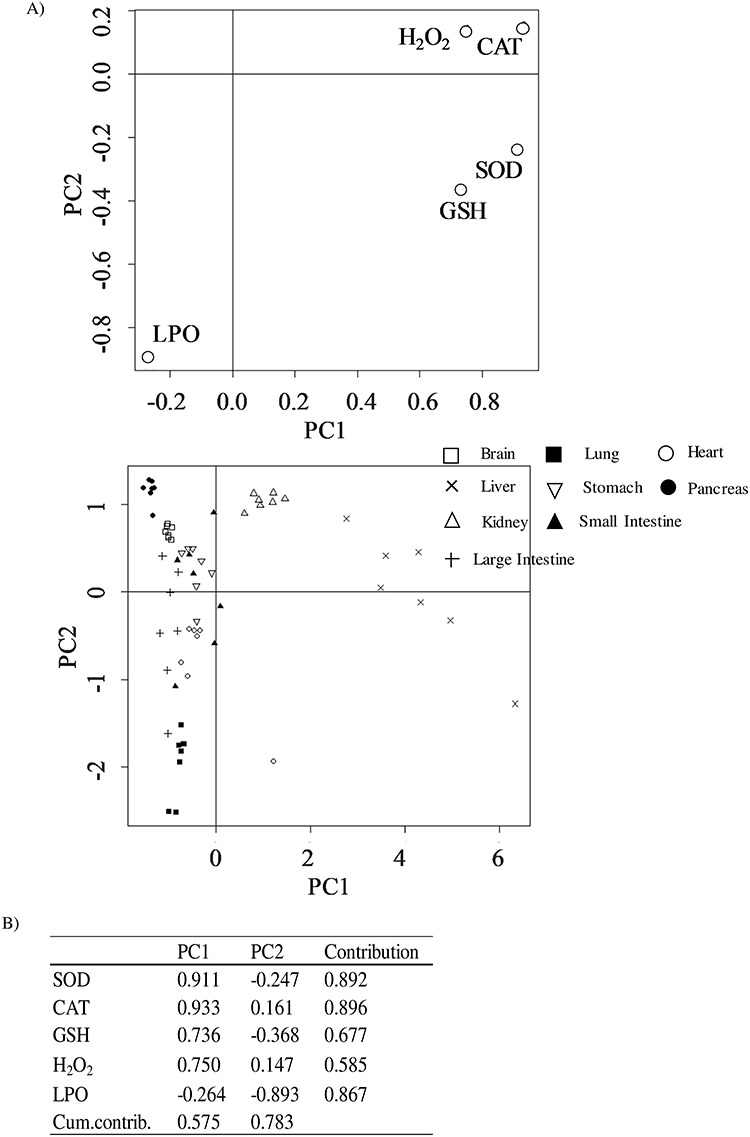
Evaluation of the redox state of different organs in sham-inhaled mice. (**A**) PCA plot representing the redox state data and (**B**) results of PCA. The contribution ratio of each indicator to each axis (PC1, PC2) is shown for each plot. Cumulative contribution (Cum. contribut.) is the ratio of the contribution of each component to the total contribution. White square, brain; black square, lung; white circle, heart; cross, liver; white inverted triangle, stomach; black circle, pancreas; white triangle, kidney; black triangle, small intestine; plus, large intestine.

To evaluate the characteristics of the redox state in each organ, PCA was performed. A scatter plot representing antioxidative functions as PC1 and oxidative stress as PC2 was obtained from the PCA of sham-inhaled mice (Fig. 1A). The contribution of SOD and CAT to PC1 and that of LPO to PC2 were substantial (Fig. 1B). The results of PC1 showed that the liver and kidney had high antioxidant capacities (Group 1). In contrast, the results of PC2 showed that LPO levels in the brain, pancreas and stomach were relatively low (Group 2), whereas LPO levels in the lungs, heart, small intestines and large intestines were relatively high (Group 3; Fig. 1A).

### Changes in SOD activity, CAT activity, t-GSH content, LPO levels and H_2_O_2_ levels in organs

**Fig. 2.**
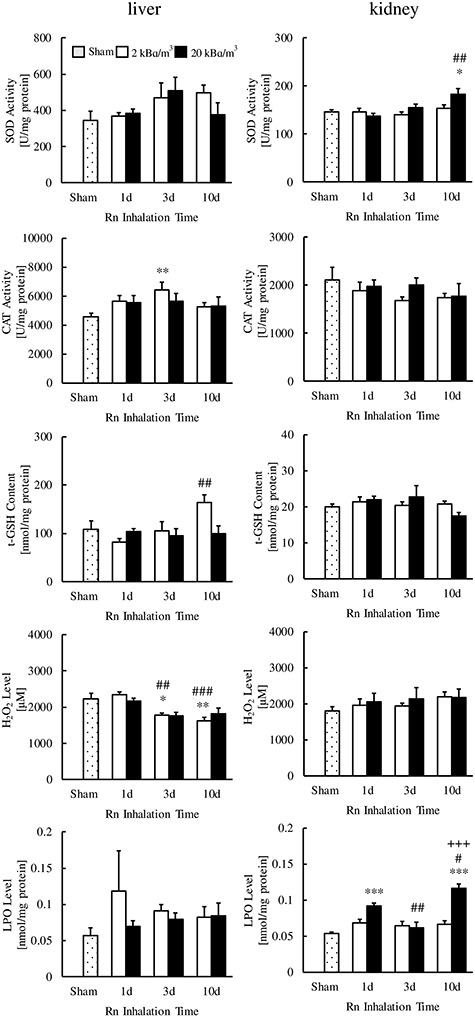
Changes in SOD activity, CAT activity, t-GSH contents, H_2_O_2_ levels and LPO levels in the liver and kidneys following radon (Rn) inhalation. The number of mice per experimental point was 6–7. ^*^P < 0.05, ^**^P < 0.01, ^*^^*^^*^P < 0.001 vs sham; ^#^*P* < 0.05, ^##^*P* < 0.01, ^###^*P* < 0.001 vs 1 day; ^+++^*P* < 0.001 vs 3 days.

**Fig. 3.**
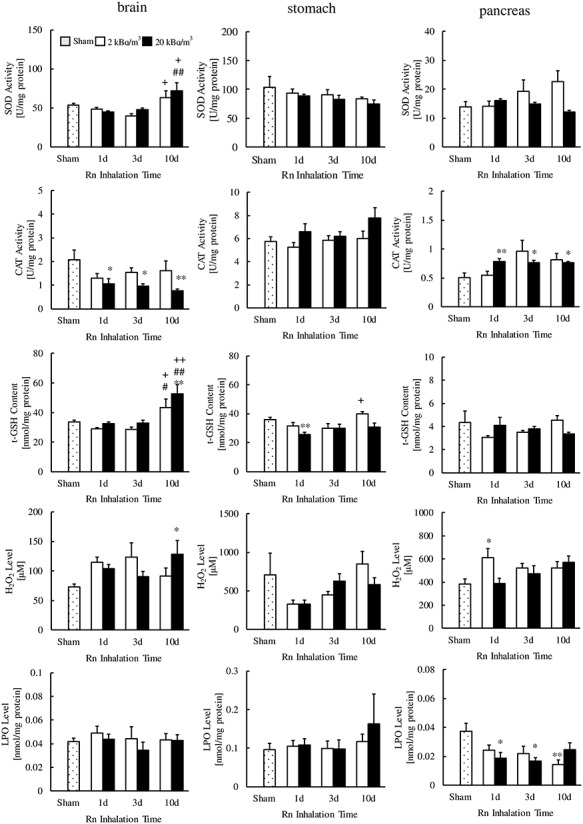
Changes in SOD activity, CAT activity, t-GSH contents, H_2_O_2_ levels and LPO levels in the brain, stomach and pancreas following radon (Rn) inhalation. The number of mice per experimental point was 4–7. ^*^P < 0.05, ^*^^*^P < 0.01 vs sham; ^#^*P* < 0.05, ^##^*P* < 0.01 vs 1 day; ^+^*P* < 0.05; ^++^*P* < 0.01 vs 3 days.

**Fig. 4.**
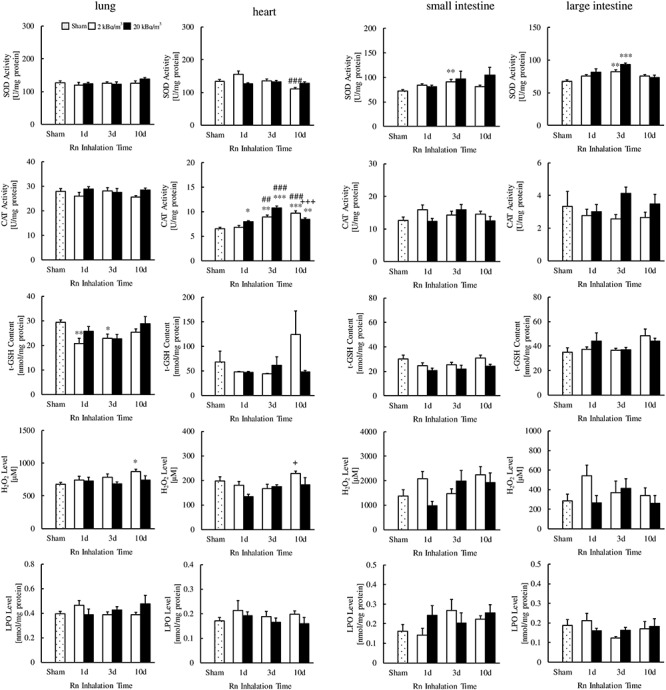
Changes in SOD activity, CAT activity, t-GSH contents, H_2_O_2_ levels and LPO levels in the lungs and heart, small intestines and large intestines following radon (Rn) inhalation. The number of mice per experimental point was 7. ^*^P < 0.05, ^*^^*^P < 0.01, ^*^^*^^*^P < 0.001 vs sham; ^##^*P* < 0.01, ^###^*P* < 0.001 vs 1 day; ^+^*P* < 0.05, ^+++^*P* < 0.001 vs 3 days.

As shown in Figs 2, 3, and 4, the SOD activities in the kidney (20 kBq/m^3^ for 10 days), small intestine (2 kBq/m^3^ for 3 days) and large intestine (2 or 20 kBq/m^3^ for 3 days) of radon-inhaled mice were significantly higher than those of the sham-inhaled mice. The CAT activities were higher in the heart (2 kBq/m^3^ for 3 or 10 days, 20 kBq/m^3^ for 1, 3 or 10 days), liver (2 kBq/m^3^ for 3 days) and pancreas (20 kBq/m^3^ for 1, 3 or 10 days) of the former than in that of the latter. However, CAT activities were lower in the brain of radon-inhaled mice (20 kBq/m^3^ for 1, 3 or 10 days) than in the brain of sham-inhaled mice.

Furthermore, radon inhalation increased the t-GSH contents in the brain (20 kBq/m^3^ for 10 days); H_2_O_2_ levels in the brain (20 kBq/m^3^ for 10 days), lung (2 kBq/m^3^ for 10 days) and pancreas (2 kBq/m^3^ for 1 day); and LPO levels in the kidney (20 kBq/m^3^ for 1 or 10 days). In contrast, radon inhalation decreased the t-GSH contents in the lung (2 kBq/m^3^ for 1 or 3 days) and stomach (20 kBq/m^3^ for 1 day), H_2_O_2_ levels in the liver (2 kBq/m^3^ for 3 and 10 days) and LPO levels in the pancreas (2 kBq/m^3^ for 10 days, 20 kBq/m^3^ for 1 or 3 days).

### Evaluation of the redox state of organs following radon inhalation

Comparative correlation analyses between radon- and sham-inhaled mice were performed to evaluate the effects of radon inhalation on the redox state of different organs in the three groups indicated in the previous section. The correlation coefficients related to GSH, H_2_O_2_ and LPO in most organ groups were changed following radon inhalation. The correlation coefficients related to LPO and H_2_O_2_ in the liver and kidney were changed following radon inhalation at a concentration of 2 kBq/m^3^ for 3 or 10 days, respectively. In addition, the correlation coefficients related to SOD in the brain, pancreas and stomach were changed following radon inhalation at a concentration of 20 kBq/m^3^ for 10 days, and those related to SOD in the lungs, heart, small intestines and large intestines were changed following radon inhalation at a concentration of 2 kBq/m^3^ for 1 day (Tables 1–3).

**Table 1:** Correlation coefficient for each indicator in the liver and kidney. ^*^P < 0.05, ^*^^*^P < 0.01, ^*^^*^^*^P< 0.001. Highlights show that radon inhalation caused changes in the indicators compared with sham irradiation

Sham (high antioxidant capacity)																	
	SOD	CAT	GSH	H_2_O_2_	LPO												
SOD	1																
CAT	0.695^*^^*^	1															
GSH	0.983^*^^*^^*^	0.726	1														
H_2_O_2_	0.165	0.395	0.298	1													
LPO	−0.337	0.015	−0.195	0.653^*^	1												
2 kBq/m^3^ 1 day						2 kBq/m^3^ 3 day						2 kBq/m^3^ 10 day					
	SOD	CAT	GSH	H_2_O_2_	LPO		SOD	CAT	GSH	H_2_O_2_	LPO		SOD	CAT	GSH	H_2_O_2_	LPO
SOD	1					SOD	1					SOD	1				
CAT	0.974^*^^*^^*^	1				CAT	0.688^*^^*^	1				CAT	0.863^*^^*^^*^	1			
GSH	0.961^*^^*^^*^	0.969^*^^*^^*^	1			GSH	0.986^*^^*^^*^	0.698^*^^*^	1			GSH	0.994^*^^*^^*^	0.892^*^^*^^*^	1		
H_2_O_2_	0.445	0.409	0.466	1		H_2_O_2_	−0.391	−0.468	−0.331	1		H_2_O_2_	−0.689^*^^*^	−0.624^*^	−0.686^*^^*^	1	
LPO	0.321	0.370	0.298	−0.023	1	LPO	0.598^*^^*^	0.495^*^	0.647^*^	0.007	1	LPO	0.097	0.163	0.083	−0.345	1
20 kBq/m^3^ 1 day						20 kBq/m^3^ 3 day						20 kBq/m^3^ 10 day					
	SOD	CAT	GSH	H_2_O_2_	LPO		SOD	CAT	GSH	H_2_O_2_	LPO		SOD	CAT	GSH	H_2_O_2_	LPO
SOD	1					SOD	1					SOD	1				
CAT	0.760^*^^*^	1				CAT	0.680^*^^*^	1				CAT	0.561^*^	1			
GSH	0.995^*^^*^^*^	0.783^*^^*^^*^	1			GSH	0.988^*^^*^^*^	0.699^*^^*^	1			GSH	0.940^*^^*^^*^	0.719^*^^*^	1		
H_2_O_2_	0.160	0.161	0.127	1		H_2_O_2_	−0.231	−0.317	−0.182	1		H_2_O_2_	−0.213	−0.386	−0.234	1	
LPO	−0.551	−0.578^*^	−0.570	−0.187	1	LPO	0.207	0.143	0.183	−0.262	1	LPO	−0.125	−0.154	−0.307	0.089	1

**Table 2:** Correlation coefficient for each indicator of pancreas, brain and stomach. ^*^P < 0.05, ^*^^*^P< 0.01, ^*^^*^^*^P < 0.001. Highlights show that radon inhalation caused changes in the indicators compared with sham irradiation

Sham (low antioxidant capacity but low LPO content)																	
	SOD	CAT	GSH	H_2_O_2_	LPO												
SOD	1																
CAT	0.828^*^^*^^*^	1															
GSH	0.706^*^^*^^*^	0.705^*^^*^^*^	1														
H_2_O_2_	−0.166	0.322	0.053	1													
LPO	0.434^*^	0.725^*^^*^^*^	0.525^*^	0.675^*^^*^^*^	1												
2 kBq/m^3^ 1 day						2 kBq/m^3^ 3 day						2 kBq/m^3^ 10 day					
	SOD	CAT	GSH	H_2_O_2_	LPO		SOD	CAT	GSH	H_2_O_2_	LPO		SOD	CAT	GSH	H_2_O_2_	LPO
SOD	1					SOD	1					SOD	1				
CAT	0.939^*^^*^^*^	1				CAT	0.926^*^^*^^*^	1				CAT	0.639^*^^*^	1			
GSH	0.842^*^^*^^*^	0.673^*^^*^^*^	1			GSH	0.706^*^^*^^*^	0.613^*^^*^	1			GSH	0.916^*^^*^^*^	0.423	1		
H_2_O_2_	−0.377	−0.160	−0.691^*^^*^^*^	1		H_2_O_2_	0.014	0.180	−0.450^*^	1		H_2_O_2_	0.111	0.621^*^^*^	−0.162	1	
LPO	0.781^*^^*^^*^	0.813^*^^*^^*^	0.579^*^^*^	−0.274	1	LPO	0.758^*^^*^^*^	0.787^*^^*^^*^	0.437	−0.083	1	LPO	0.672^*^^*^	0.836^*^^*^^*^	0.492^*^	0.342	1
20 kBq/m^3^ 1 day						20 kBq/m^3^ 3 day						20 kBq/m^3^ 10 day					
	SOD	CAT	GSH	H_2_O_2_	LPO		SOD	CAT	GSH	H_2_O_2_	LPO		SOD	CAT	GSH	H_2_O_2_	LPO
SOD	1					SOD	1					SOD	1				
CAT	0.882^*^^*^^*^	1				CAT	0.841^*^^*^^*^	1				CAT	0.343	1			
GSH	0.622^*^^*^	0.323	1			GSH	0.736^*^^*^^*^	0.386	1			GSH	0.890^*^^*^^*^	0.048	1		
H_2_O_2_	−0.076	0.281	−0.574^*^^*^	1		H_2_O_2_	0.234	0.524^*^	−0.249	1		H_2_O_2_	−0.503^*^	0.420	−0.699^*^^*^	1	
LPO	0.842^*^^*^^*^	0.768^*^^*^^*^	0.383	−0.076	1	LPO	0.629^*^^*^	0.735^*^^*^^*^	0.323	0.063	1	LPO	0.338	0.569^*^	0.166	0.255	1

**Table 3:** Correlation coefficient for each indicator of the lung, small intestine, large intestine, and heart. ^*^P < 0.05, ^*^^*^P < 0.01, ^*^^*^^*^P < 0.001. Highlights show that radon inhalation caused changes in the indicators compared with sham irradiation

Sham (high LPO content but low antioxidant capacity)																	
	SOD	CAT	GSH	H_2_O_2_	LPO												
SOD	1																
CAT	0.408^*^	1															
GSH	0.172	−0.206	1														
H_2_O_2_	−0.314	0.320	−0.231	1													
LPO	0.413^*^	0.680^*^^*^^*^	−0.157	0.012	1												
2 kBq/m^3^ 1 day						2 kBq/m^3^ 3 day						2 kBq/m^3^ 10 day					
	SOD	CAT	GSH	H_2_O_2_	LPO		SOD	CAT	GSH	H_2_O_2_	LPO		SOD	CAT	GSH	H_2_O_2_	LPO
SOD	1					SOD	1					SOD	1				
CAT	0.131	1				CAT	0.402^*^	1				CAT	0.657^*^^*^^*^	1			
GSH	0.394^*^	−0.734^*^^*^^*^	1			GSH	0.221	−0.680^*^^*^^*^	1			GSH	0.093	−0.210	1		
H_2_O_2_	−0.446^*^	0.322	−0.594^*^^*^^*^	1		H_2_O_2_	−0.367	0.326	−0.653^*^^*^^*^	1		H_2_O_2_	−0.286	0.287	−0.271	1	
LPO	0.190	0.488^*^	−0.392^*^	−0.135	1	LPO	0.257	0.744^*^^*^^*^	−0.551^*^^*^	0.323	1	LPO	0.477^*^	0.779^*^^*^^*^	−0.231	0.140	1
20 kBq/m^3^ 1 day						20 kBq/m^3^ 3 day						20 kBq/m^3^ 10 day					
	SOD	CAT	GSH	H_2_O_2_	LPO		SOD	CAT	GSH	H_2_O_2_	LPO		SOD	CAT	GSH	H_2_O_2_	LPO
SOD	1					SOD	1					SOD	1				
CAT	0.442^*^	1				CAT	0.194	1				CAT	0.504^*^^*^	1			
GSH	0.286	−0.503^*^^*^	1			GSH	0.182	−0.294	1			GSH	−0.061	−0.511^*^^*^	1		
H_2_O_2_	−0.258	0.473^*^	−0.691^*^^*^^*^	1		H_2_O_2_	−0.314	0.205	−0.319	1		H_2_O_2_	−0.006	0.239	−0.658^*^^*^^*^	1	
LPO	0.177	0.655^*^^*^^*^	−0.444^*^	0.378^*^	1	LPO	−0.023	0.770^*^^*^^*^	−0.314	0.047	1	LPO	0.190	0.686^*^^*^^*^	−0.520^*^^*^	0.128	1

## DISCUSSION

Several studies showing the effects of radon therapy have reported the activation of SOD activities in different organs [9–14, 20]. Moreover, radiation has been shown to induce reactive oxygen species (ROS), with the yield of ROS varying depending on the linear energy transfer [21]. The antioxidant system in the body can also produce ROS. For example, the scavenging activity of SOD involves the conversion of the superoxide anion radical (O_2_^−^) into H_2_O_2_ [22]. However, H_2_O_2_ is detoxified by CAT and GSH peroxidase (GPx), which are the two most important enzymes that regulate intracellular H_2_O_2_ levels in biological systems [23]. The former is thought to play a major role in the excessive production of H_2_O_2_ [24, 25]. GSH directly reacts with ROS, and GPx catalyzes the destruction of H_2_O_2_ and hydroperoxide [26]. Because undecomposed excessive H_2_O_2_ can lead to the production of hydroxyl radicals by the Fenton reaction, CAT and GSH play important roles in protection against ROS. Therefore, evaluation of the redox state and the balance among antioxidant-associated substances, such as SOD, CAT, t-GSH, LPO and H_2_O_2_, is more important than evaluating individual indicators.

In the current study, the above antioxidants were considered when determining the effects of radon inhalation on different organs. In sham-inhaled mice, the organs were classified into three groups based on their redox state. Furthermore, estimation of the correlation coefficients in each group revealed that compared to those of the sham group, the correlation coefficients related to GSH, H_2_O_2_ and LPO for most groups were changed following radon inhalation. This result suggested that radon inhalation altered oxidative stress-related indicators and that t-GSH played an important role in maintaining the redox state of organs. In addition, correlation coefficients related to SOD in Groups 2 and 3 were also changed, indicating that SOD may have critical roles in complementing low antioxidant capacity. The response to radon varied depending on the redox state in organs. In addition, the SOD-related correlations changed in organs with low antioxidant capacity but not in those with high antioxidant capacity. Furthermore, the absorbed doses for different organs were almost identical (data not shown) and within the same range, as reported earlier [27]. Therefore, the organs evaluated in this study likely produced almost the same amount of ROS following radon inhalation, and the observed differences in the effects of radon inhalation on different organs could be attributed to differences in their total antioxidant capacities. Specifically, organs having lower antioxidant capacity showed an altered redox state, which may have induced oxidative stress in organs following radon inhalation.

Moderate oxidative stress induced by radon results in Mn-SOD production [15], whereas excessive stress induced by high-dose irradiation decreases antioxidative functions [28]. Thus, to promote the beneficial therapeutic effects of radon therapy, elucidation of the appropriate dose and duration is essential. In this study, a comparison of the effects of low and high radon concentrations revealed significant negative correlations between antioxidant and H_2_O_2_ levels in the organs of Group 1 subjected to a low-dose radon inhalation (2 kBq/m^3^ for 10 days) but no significant changes in the high-dose group (20 kBq/m^3^ for 10 days). These findings demonstrating a dose-dependent effect could be used to develop therapeutic strategies targeting individual organs. For example, an inhalation dose of 2 kBq/m^3^ for 10 days could be the optimum conditions to prevent oxidative stress in the liver because this dose reduced H_2_O_2_ levels in the liver.

Furthermore, the changes observed in SOD-related correlations of Group 2 organs exposed to 20 kBq/m^3^ for 10 days indicated the effects of radon therapy duration. Consistent with this finding, an earlier study reported similar temporal effects of low-dose X-irradiation on SOD activity [29]. Although the underlying mechanisms of these effects have not been explored, the delayed production of ROS in response to X-irradiation could be an important factor [30]; further studies are needed to confirm this notion.

Antioxidants, such as SOD, have critical roles in inhibiting ischemia–reperfusion injuries in the liver [31]. Therefore, we speculate that radon therapy could also inhibit ischemia–reperfusion injuries in the liver. However, the long duration required for effective radon therapy could be a limitation for its clinical application. As shown in our previous study, the combination of radon inhalation with antioxidants, such as vitamin C and vitamin E, could be an ideal therapeutic strategy for ischemia–reperfusion injuries in the liver [32].

To date, only a few reports have revealed that radon inhalation increases antioxidative functions in the heart. In the current study, radon inhalation significantly increased CAT activities in the heart. These findings suggest that cardiac diseases induced by oxidative stress may be inhibited by radon inhalation. However, further studies are needed to clarify the positive effects of radon inhalation.

## CONCLUSIONS

In conclusion, we found that radon inhalation altered the correlation coefficients of oxidative stress-related indicators and t-GSH. In addition, we showed that SOD played an important role in determining the redox state of tissues with low antioxidant capacities. These findings suggest that radon inhalation can change the redox state in organs; however, this characteristic can vary depending on the redox state. The findings of this study can be extended to investigate the differences between the therapeutic radon concentration used in the Misasa and Montana studies. The insights obtained from this study on the dose and duration dependency of the redox state may help develop therapeutic strategies targeting individual organs. However, the results obtained here are based on correlations; therefore, further studies are needed to clarify the causal relationships and underlying mechanisms.

## CONFLICT OF INTEREST

None declared.

## FUNDING

This work was supported by JAEA Nuclear Energy S&T and Human Resource Development Project through Concentrating Wisdom (grant no. JPJA18B18072098).
