# Erratum to: Evaluation of the redox state in mouse organs following radon inhalation

**DOI:** 10.1093/jrr/rrab072

**Published:** 2021-07-21

**Authors:** Takahiro Kataoka, Norie Kanzaki, Akihiro Sakoda, Hina Shuto, Junki Yano, Shota Naoe, Hiroshi Tanaka, Katsumi Hanamoto, Hiroaki Terato, Fumihiro Mitsunobu, Kiyonori Yamaoka

**Affiliations:** 1 Graduate School of Health Sciences, Okayama University, 5-1 Shikata-cho 2-chome, Kita-ku, Okayama-shi, Okayama 700-8558, Japan; 2 Ningyo-toge Environmental Engineering Center, Japan Atomic Energy Agency, 1550 Kamisaibara, Kagamino-cho, Tomata-gun, Okayama 708-0698, Japan; 3 Advanced Science Research Center, Okayama University, 5-1 Shikata-cho 2-chome, Kita-ku, Okayama-shi, Okayama 700-8558, Japan; 4 Graduate School of Medicine Dentistry and Pharmaceutical Sciences, Okayama University, 5-1 Shikata-cho 2-chome, Kita-ku, Okayama-shi, Okayama 700-8558, Japan

Due to a technical error, the paper 10.1093/jrr/rraa129, which was first published in Journal of Radiation Research Volume 62 Issue 2 was mistakenly republished in Journal of Radiation Research Volume 62 Issue 3. Upon identification of this error, 10.1093/jrr/rraa129 was removed from Issue 3, where it should not have appeared. It remains in its correct place of first publication - Volume 62 Issue 2.

Page numbers were assigned to 10.1093/jrr/rraa129 within Volume 62 Issue 3. When 10.1093/jrr/rraa129 was removed from Volume 62 Issue 3 no changes were made to the page numbers of the papers that followed. The page numbers: 390-400, which were assigned to 10.1093/jrr/rraa129 when it mistakenly appeared in Volume 62 Issue 3, are now left intentionally blank within the volume.

